# Return of non-ACMG recommended incidental genetic findings to pediatric patients: considerations and opportunities from experiences in genomic sequencing

**DOI:** 10.1186/s13073-022-01139-2

**Published:** 2022-11-21

**Authors:** Kevin M. Bowling, Michelle L. Thompson, Melissa A. Kelly, Sarah Scollon, Anne M. Slavotinek, Bradford C. Powell, Brian M. Kirmse, Laura G. Hendon, Kyle B. Brothers, Bruce R. Korf, Gregory M. Cooper, John M. Greally, Anna C. E. Hurst

**Affiliations:** 1 HudsonAlpha Institute for Biotechnology, Huntsville, AL 35806 USA; 2 Department of Pathology and Immunology, Washington University School of Medicine, St. Louis, MO 63110 USA; 3 HudsonAlpha Clinical Services Lab, LLC, HudsonAlpha Institute for Biotechnology, Huntsville, USA; 4 Department of Pediatrics, Baylor College of Medicine, Houston, TX 77030 USA; 5 Department of Pediatrics, University of California, San Francisco, CA 94158 USA; 6 Department of Genetics, University of North Carolina at Chapel Hill, Chapel Hill, NC 27599 USA; 7 Department of Pediatrics, University of Mississippi Medical Center, Jackson, MS 39216 USA; 8 Norton Children’s Research Institute Affiliated with UofL School of Medicine, Louisville, KY 40202 USA; 9 Department of Genetics, University of Alabama at Birmingham, Birmingham, AL 25294 USA; 10 Department of Genetics, Albert Einstein College of Medicine, Bronx, NY 10461 USA

**Keywords:** Incidental findings, Genomics, Genetic testing, Pediatrics, Disease severity, Personal utility, Actionability, Penetrance, Age of onset

## Abstract

**Background:**

The uptake of exome/genome sequencing has introduced unexpected testing results (incidental findings) that have become a major challenge for both testing laboratories and providers. While the American College of Medical Genetics and Genomics has outlined guidelines for laboratory management of clinically actionable secondary findings, debate remains as to whether incidental findings should be returned to patients, especially those representing pediatric populations.

**Methods:**

The Sequencing Analysis and Diagnostic Yield working group in the Clinical Sequencing Evidence-Generating Research Consortium has collected a cohort of pediatric patients found to harbor a genomic sequencing-identified non-ACMG-recommended incidental finding. The incidental variants were not thought to be associated with the indication for testing and were disclosed to patients and families.

**Results:**

In total, 23 "non-ACMG-recommended incidental findings were identified in 21 pediatric patients included in the study. These findings span four different research studies/laboratories and demonstrate differences in incidental finding return rate across study sites. We summarize specific cases to highlight core considerations that surround identification and return of incidental findings (uncertainty of disease onset, disease severity, age of onset, clinical actionability, and personal utility), and suggest that interpretation of incidental findings in pediatric patients can be difficult given evolving phenotypes. Furthermore, return of incidental findings can benefit patients and providers, but do present challenges.

**Conclusions:**

While there may be considerable benefit to return of incidental genetic findings, these findings can be burdensome to providers and present risk to patients. It is important that laboratories conducting genomic testing establish internal guidelines in anticipation of detection. Moreover, cross-laboratory guidelines may aid in reducing the potential for policy heterogeneity across laboratories as it relates to incidental finding detection and return. However, future discussion is required to determine whether cohesive guidelines or policy statements are warranted.

**Supplementary Information:**

The online version contains supplementary material available at 10.1186/s13073-022-01139-2.

## Background

As clinical and research laboratories employ more genomic testing (exome/genome sequencing, ES/GS) in pediatric patients, the detection of incidental findings (IF) is becoming much more common. IFs are genetic findings that have medical relevance but that are not related to the indication for testing and not intentionally sought during analysis [1, 2]. This definition is distinct from secondary findings (SF) [1], which, while like IFs are not related to the indication for testing, they are identified as a result of a deliberate search for medically relevant variants [1–3].

US guidelines have been established for returning a set list of actionable SFs for both children and adults [3–5] and many laboratories have incorporated these into their standard protocols and consent documentation. Several international groups (European Society for Human Genetics (ESHG), the Canadian College of Medical Geneticists (CCMG), and EuroGentest), all recommend targeted data analysis to reduce SF and/or IF identification, but suggest laboratories provide patients with the option to receive such findings [6–8]. Both ESHG and EuroGentest allow laboratories to decide whether or not IFs shall be returned but call for use of well-defined protocols [7, 8]. The CCMG does not advocate for searching for SF and that only some IFs should be returned. Thus, they have established guidelines for returning IFs to adults (e.g., no VUS nor low-penetrance findings) and pediatric patients (e.g., highly penetrant childhood onset; if adult-onset, only at parent’s request and prevention of serious harm to parent or family member) [6]. No specific policies pertaining to IFs have been adopted by US governing bodies, although guidance may be warranted as expanded ES/GS testing increases potential for IF detection.

Predictive genetic testing in pediatric patients has traditionally been discouraged, especially for cases where prevention, management, or treatment options are unavailable (not “clinically actionable”) or are related to adult-onset conditions about which the child should be allowed to, when they reach adulthood, make their own decisions. Although some pediatric genomic studies have limited return of results to a narrowed panel of disease-relevant genes (based on disease phenotype) to reduce the likelihood of detecting IFs, these types of results may still emerge during analysis [9]. Prior publications have also identified the hypothetical risks and benefits of returning IFs and speculate about whether such findings provide personal utility (“best interests” or “benefit to families” [10–12]). Meanwhile, it is becoming increasingly evident that young patients and their families can successfully manage knowledge obtained from predictive testing, and as such, return of genetic findings not related to the indication for testing has become more acceptable [13–15]. A recent publication by Garrett and colleagues [16] found that predictive testing in children is viewed by many as ethically permissible if the potential benefits outweigh the unrealized harms.

There are several core issues associated with return of IFs in pediatric patients. These issues include uncertainty of disease onset (penetrance, variable expressivity), severity of the condition, age of onset, clinical actionability, and personal utility [17]. Moreover, determination of whether an ES/GS-identified genetic variant is truly unrelated to the indication for testing can be complicated for pediatric patients when phenotype assessment is limited by developmental stage (e.g., in newborns), or when limited phenotype information is available to the testing laboratory. Very young pediatric patients represent those most likely to have evolving phenotypes, and laboratories must rely on documented clinical histories and phenotypes provided, which could quickly change or become outdated. Therefore, masking/filtering out variants not associated with current phenotypes could lead to a missed genetic diagnosis. Finally, it can also be difficult to determine if a finding is truly unrelated to the patient’s current clinical presentation given limitations in defining the expected phenotypes for any specific genetic condition.

The Clinical Sequencing Evidence-Generating Research Consortium (CSER), funded by the National Human Genome Research Institute (NHGRI), the National Cancer Institute (NCI), and the National Institute on Minority Health and Health Disparities (NIMHD), is a national multi-site research program focused on engaging traditionally underrepresented populations in genomic research and implementing genomic sequencing into the clinical care of diverse and medically underserved populations. This study included three CSER-funded studies as well as one other research study at a CSER-participating institution, and together, the four studies have identified 23 IFs across 21 pediatric patients. Our laboratories have taken a case-by-case approach in identifying, interpreting, and returning IFs to pediatric patients and families, and this approach varied by site. In doing so, we have gained insight into the types of IFs that may be discovered when using genomic sequencing in younger patients, their potential for benefit, and the considerations that must be taken when deciding whether to return them to patients.

In this report, we highlight cases that demonstrate direct benefit of IF return, discuss associated challenges, and underscore lessons learned. This information may inform other laboratories, researchers, and genetics providers who are conducting or ordering genomic testing for pediatric patients.

## Methods

SouthSeq [18], KidsCanSeq, P^3^EGS [19], and COAGS are research studies aimed at determining the benefit of using genomic sequencing (ES/GS) in pediatric patients to identify genetic variation underlying disease etiology (Table 1). SouthSeq, KidsCanSeq, and P^3^EGS are funded by the NIH as part of the CSER consortium.

**Table 1:** Study sites that identified medically relevant incidental genetic findings in pediatric patients

Study	Aims
SouthSeq	SouthSeq is performing genome sequencing to diagnose infants suspected to have genetic disorders. The study is also running a clinical trial to develop and test different return of results mechanisms to expand access to genetic testing to diverse, underserved communities across the Southeastern US.
KidsCanSeq	The Baylor College of Medicine KidsCanSeq Study aims to assess the utility of genome-scale testing, compared with more targeted methods, in diverse pediatric cancer patient populations and diverse healthcare settings in Texas.
P^3^EGS	The UCSF Program in Prenatal and Pediatric Genome Sequencing (P^3^EGS) is studying the utility of exome sequencing as a tool to diagnose infants and children with serious developmental disorders and provide genetic information to parents when a prenatal study reveals a fetus with a structural anomaly.
COAGS	The Children’s of Alabama Genome Sequencing study (COAGS) is conducting genome sequencing for pediatric patients with rare disease phenotypes and is working to identify clinical indicators that may predict the likelihood of genetic diagnosis using genome testing.

Discussion in the Sequencing Analysis and Diagnostic Yield Working Group (SADY) working group revealed that several CSER studies, as well as one external study (COAGS), were identifying medically relevant IFs in genes not on the ACMG secondary findings gene list (3-5) in young patients, in addition to searching for SFs in genes included on the ACMG list.

In this context, IFs are variants that are: not associated with the indication for testing; classified as pathogenic or likely pathogenic according to ACMG-AMP guidelines [20]; not specifically sought during genomic analysis and therefore not in genes on the ACMG SF gene lists (3-5); in genes leading to childhood or adolescent-onset disorders for which the patient may be pre-symptomatic at time of testing or associated with adult-onset disease risk (high or low risk); and considered to be potentially clinically relevant. The discussion among SADY working group members focused on the challenges and opportunities associated with these types of findings, whether such findings should be returned, and the implications of these types of findings for patients, families, testing laboratories, and providers. Most importantly, the group discussed potential for benefit and burden (e.g., potential to improve outcomes, the risk to patients, clinical management by providers) to both patients/families and providers.

### SouthSeq

The institutional review board at the University of Alabama at Birmingham (IRB-300000328) approved and monitored the study. All individual-level data were de-identified to the research team. The authors received and archived written patient consent to publish individual data.

Participant infants were enrolled at one of five clinical sites, namely the University of Alabama at Birmingham/Children’s of Alabama (Birmingham, AL), University of Mississippi Medical Center (Jackson, MS), Woman’s Hospital (Baton Rouge, LA), Norton Children’s Hospital/University of Louisville (Louisville, KY), and Children’s Hospital New Orleans (New Orleans, LA). The consent of at least one parent/legal guardian was required for study participation. To be included in the study, a participant had to be inpatient, in the first 12 months of life, and exhibit a pattern of congenital anomalies consistent with a genetic disorder of unknown etiology [18]. Parents also had the option to consent to receive secondary findings, broadly defined in the consent documentation as genetic variation associated with increased disease risk (ACMG SF gene lists (3-5)). At time of consent, no distinction was made between secondary and incidental findings. No incidental findings were returned to families that chose to opt out of ACMG secondary findings.

Peripheral or cord blood samples collected in EDTA tubes were sent to the HudsonAlpha Clinical Services Laboratory (CSL) for DNA extraction (QIAsymphony) and storage. Sequencing libraries were constructed from genomic DNA using the CSL’s custom genome library preparation protocol. DNA library fragments were sequenced from both ends (paired) with a read length of 150 base pairs using the Illumina HiSeq X or NovaSeq 6000 (Illumina) with a targeted mean coverage depth of 30× and >80% of bases covered at 20×. Sequence reads were aligned to GRCh38 using the DRAGEN Bio-IT platform (Illumina, [21] or the Sentieon implementation [22]) of the BWA-MEM. SNVs/indels were called using DRAGEN and GATK [23] or Strelka [24]. CNVs were called using DELLY [25], ERDS [26], Manta [27], and CNVnator [28]. Identified SNVs/indels/CNVs were annotated, filtered, and manually curated. Variants were classified in accordance with ACMG-AMP guidelines [20]. Because analysis was conducted using a research protocol, variants deemed to be returnable were clinically tested (via Sanger or array sequencing) to confirm variant presence, determine variant inheritance (when parent samples were available), and generate a report with clinical interpretation [18].

### KidsCanSeq

The study was approved by the Baylor College of Medicine Institutional Review Board (protocol H-42376). Participants were enrolled at six Texas KidsCanSeq sites: Texas Children’s Hospital (Houston, TX), Vannie E. Cook, Jr. Children’s Cancer and Hematology Clinic (McAllen, TX), Cook Children’s Medical Center (Fort Worth, TX), Children’s Hospital of San Antonio, University of Texas MD Anderson Cancer Center, and University of Texas Health Science Center at San Antonio. Informed consent was obtained from at least one parent/legal guardian and the patient provided assent. Parents were provided the option to opt out of receiving the non-cancer genes on the ACMG SFv2.0 list [3]. In KidsCanSeq, no distinction between secondary and incidental findings was made at time of consent. No incidental findings were returned to families who opted out of secondary finding return. The study enrolled patients <18 years of age with diagnoses of CNS and non-CNS solid tumors, lymphomas, and rare histiocytic disorders.

Germline ES for KidsCanSeq patients was performed in the Human Genome Sequencing Center – Clinical Laboratory (HGSC-CL) at Baylor College of Medicine and reported by Baylor Genetics as previously described [29] including library construction, exome capture by VCRome, version 2.1 supplemented with PKv1 and PKv2 probes for under-covered regions [30] (Roche NimbleGen), and paired-end sequencing on HiSeq 2000/2500 (Illumina Inc). When provided, parental samples were analyzed for variants detected in the patient’s sample. Variants in any cancer susceptibility gene were reported and classified in accordance with ACMG-AMP guidelines [3, 20]. In parallel, targeted germline testing with a pediatric cancer-focused panel for mutations and gene-level copy number alterations was also performed at Texas Children’s Hospital.

### P^3^EGS

Patients were enrolled in the Prenatal and Pediatric Genome Sequencing (P^3^EGS) study at the UCSF Benioff Children’s Hospital, the Betty Irene Moore Women’s Hospital, the Zuckerberg San Francisco General Hospital (ZSFH), UCSF Benioff Children’s Hospital in Oakland and the Community Medical Center in Fresno. The consent of at least one parent/legal guardian was required for study participation. All patients were provided with the option to receive secondary findings as per the ACMG-AMP guidelines [3–5]. Eligibility for pediatric patients included a diagnosis of multiple congenital anomalies (MCA), intellectual disability (ID), metabolic disease, epilepsy, neurodegenerative disease/cerebral palsy (CP), and encephalopathy. Prenatal eligibility criteria included one or more fetal structural abnormalities, an unexplained disorder of fetal growth, and nonimmune hydrops or a single fetal effusion.

ES was performed (as described in Mendelson and colleagues [30]) as a clinical test using a bioinformatics pipeline developed by the Institute for Human Genetics (IHG) at UCSF. The Ingenuity Variant Analysis (IVA, Qiagen) program was used to filter out likely benign variants and to analyze the proband for candidate *de novo*, homozygous, compound heterozygous and inherited heterozygous variants that were possibly disease causing. A confidence filter, common variant filter, predicted deleterious filter, custom filters (elimination of common variants ~3 or more alleles from 80 geographically diverse controls- and pseudo-autosomal regions) were applied in a step-wise fashion. The UCSF bioinformatics pipeline utilized five different genotype callers for variant calling. Human Gene Mutation Database-Professional (HGMD-Pro), ClinVar, and Online Mendelian Inheritance in Man (OMIM) databases were evaluated both for gene-specific variants and gene-disease relationships. PubMed, PubMed Central, and Google Scholar were also used when no well-defined gene-disease relationship was established in HGMD-Pro and OMIM, and if these databases did not include the gene-specific variant identified after filtering as described above. Findings were evaluated using the published ACMG-AMP criteria for variant calling [20].

The possibility for detection of secondary genetic findings was explained to study participants at the time of consent. Potential for detection of incidental findings was not specifically mentioned during the consent process. No incidental findings were returned to families that chose to opt out of ACMG secondary findings.

### COAGS

The Children’s of Alabama Genome Sequencing Study (COAGS) was approved and monitored by the Institutional Review Board at the University of Alabama at Birmingham (IRB-170314004). Eligible participants were recruited from the pediatric population (ages 0–21 years) at Children’s of Alabama (COA) who had a likely genetic health condition which remained undiagnosed despite diagnostic attempts from their referring physician (who must be a COA pediatrician or subspecialist). The consent of at least one parent/legal guardian was required for study participation. Consent (if >14) or assent (if 7–14) was obtained from the pediatric participant, if they were developmentally capable. Peripheral blood from the patient was sent to the HudsonAlpha CSL, with samples from biological relatives also collected for cascade testing to determine inheritance. The HudsonAlpha CSL workflow employed for COAGS mirrored that described above for SouthSeq. COAGS participants chose to opt in or opt out of any or all of the following categories of incidental/secondary findings at time of study enrollment: untreatable childhood disorders, treatable adulthood disorders (including ACMG SF gene lists (3-5)), untreatable adulthood disorders, carrier of a condition, and pharmacogenomics.

## Results

Across the four pediatric genomics research studies, we describe 23 incidental findings in 19 different genes (Table 2, Additional file 1: Table S1) that met our definition of an IF (see the “Methods” section). In total, IFs were identified across 21 pediatric patients with ages ranging from early infancy to late teens; one IF was identified during prenatal testing. All IF variants were returned to patients/families. Moreover, across all four study populations, variants considered returnable as IFs were found in a non-trivial percentage of tested individuals (0.93%, 21/2246 individuals tested via ES/GS). Further breakdown demonstrates significant variability in IF rates across sites: 8.0% IF rate in COAGS (11/137); 0.47% in P^3^EGS (4/845); 0.78% in SouthSeq (5/638); and 0.16% in KidsCanSeq (1/626).

**Table 2:** Incidental genetic findings and outcomes identified across four study sites conducting genomic testing for pediatric patients

Patient ID	Study site	Age	Sex	Indication for testing	Gene	Variant effect	Disease association (mode of inheritance)	Variant origin	Disease age of onset	Outcome/challenges
1	SouthSeq	14 days	M	FTT, renal cyst, developmental hip dysplasia, PFO, retrognathia, possible intracardiac tumors	*ABCD1*	p.Arg617Cys	Adrenoleukodystrophy (XLR)	Mat	Childhood to adulthood	Post RoR testing revealed increased VLCFA levels suggestive of adrenoleukodystrophy diagnosis; empowered family and clinicians for additional testing/clinical management
2	COAGS	4 years	M	Dysmorphic features, pseudoform cleft lip, coloboma	*ANLN*	p.Arg431Cys	Focal segmental glomerulosclerosis (AD)	ND	Childhood to adulthood	Referred to nephrogenetics clinic for further evaluation. Appointment has not yet occurred
3	COAGS	3 years	F	Hydrocephalus, VSD, cleft palate, DD	*ATM*	p.Val2716Ala	Breast cancer susceptibility (AD); Ataxia-telangiectasia (AR)	ND	Adulthood	Mother requested appointment for cancer genetic counseling and testing, as there is a family history of a maternal grandmother with breast cancer in her 40s. Mother did not keep the appointment and attempt to reschedule were unsuccessful
4	COAGS	8 years	M	Hirschprung disease, ID, obesity	*ATM*	p.Lys468fs	Breast cancer susceptibility (AD); Ataxia-telangiectasia (AR)	ND	Adulthood	At RoR discussed NCCN guidelines for screening in adulthood. Offered maternal testing for the variant (father not available), but mother declined. There is no family history of cancer in either side of family
5	KidsCanSeq	8 years	M	Medulloblastoma	*ATM*	p.Asp2959fs	Breast cancer susceptibility (AD); Ataxia-telangiectasia (AR)	Pat	Adulthood	Returned as a primary finding due to nature of study design (see the “Methods” section); Tumor type has no known association with *ATM*. Given that there was no paternal family history of cancer, no screening was initiated for proband
6	SouthSeq	19 days	M	VACTERL-related features	*CACNA1A*	p.Arg1545Ter	Episodic ataxia (AD); Epileptic encephalopathy (AD)	Pat^a^	Childhood to adulthood; Birth or early infancy	Post RoR revealed father exhibited symptoms that overlap features reported for *CACNA1A*- and *PMP22*-related disorders; Father declined referral to neurology
6	SouthSeq	19 days	M	VACTERL-related features	*PMP22*, others	17p12 deletion	Hereditary neuropathy (AD)	Pat^a^	Childhood to adolescence	--
7	COAGS	5 months	F	Failure to thrive, macroglossia, central and obstructive apnea	*CHEK2*	p.Thr367fs	Cancer predisposition (AD)	ND	Adulthood	Mother requested testing for the *CHEK2* variant and is negative. Father later reported additional cancer family history that was not initially disclosed at first visit or at initial RoR. He met with genetic counseling, ordered commercial *CHEK2* testing, but later canceled the order due to out-of-pocket cost. He was then provided with information about low/no-cost commercial testing, but it is unclear if he completed testing
8	P3EGS	Prenatal	F	Fetal heart defect	*CHEK2*	p.Arg137Ter	Cancer predisposition (AD)	Mat	Adulthood	Mother referred to genetics; lost to follow up
9	COAGS	4 years	F	DD, esotropia, periventricular heterotopia	*SLC3A1*	c.1500+1G>T	Cystinuria (AD, AR)	ND	Childhood to adulthood	Discussed signs and symptoms of the condition at results and will clinically monitor; No family history of symptoms
10	P3EGS	14 months	F	Tracheoesophageal fistula, tethered cord, hypoplastic thumb, microcephaly, small size	*COL4A5*	p.Gly1116Val	Alport syndrome (XLD)	Pat^a^	Infancy to adulthood	This variant was not originally formally reported out to the family due to the incidental nature of the finding. However, it was later determined that the father of the proband has a personal history of Alport syndrome and therefore the study requested that the lab look at Alport-associated genes. It was determined that the proband harbored a pathogenic variant in COL4A5 associated with X-linked dominant Alport syndrome. This result was then returned to the family and it was recommended that the proband be followed up with nephrology.
11	SouthSeq	3 days	M	Subcutaneous mass suggestive of lipoma, lymph node or resolving hematoma; maternal family history of having "bumps all over the skin, sometimes painful"	*FLCN*	17p11.2 deletion	Birt-Hogg-Dube (BHD) syndrome (AD)	Pat	Adulthood	Originally returned as a primary finding; post RoR, event was clinically validated and determined to be paternally inherited; ultimately classified as an incidental finding. Clinicians are deferring BHD management/surveillance until adulthood
12	P3EGS	17 months	F	Bilateral thumb hypoplasia with family history of Cavanagh syndrome	*HFE*	p.Cys282Tyr	Hemochromatosis (AR)	Biparental	Adulthood	Parents had difficulty understanding the implications of this unanticipated result and the timing as any effects due to pathogenic variants in *HFE* are not anticipated until adult life
12	P3EGS	17 months	F	Bilateral thumb hypoplasia with family history of Cavanagh syndrome	*HFE*	p.His63Asp	Hemochromatosis (AR)		Adulthood	--
13	COAGS	5 years	M	DD, epilepsy, autism	*HMBS*	p.Arg116Trp	Acute Intermittent Porphyria (AD)	Mat^a^	Adolescence to adulthood	Obtained screening urine studies for the patient (pending at specialty lab). Mother requested genetic testing and is positive and referred to GI specialist in porphyria. At RoR discussion, mother endorsed symptoms of porphyria and years of GI scopes/studies which have been non-diagnostic
14	COAGS	15 years	M	Tremor, autism, hypernasal speech, congenital heart defect	*HOXB13*	p.Gly84Glu	Hereditary prostate cancer (AD)	ND	Adulthood	Mother reported that she shared copies of the child's report with both maternal and paternal relatives as it is unknown if/from whom the variant was inherited
15	P3EGS	3 years	F	ID/NDD	*MC4R*	p.Ile269Asn	Obesity (AD)	Pat^a^	Infancy	Paternal family members exhibit obesity
16	COAGS	6 years	M	DD, failure to thrive, dysmorphic features	*MFN2*	p.Arg707Trp	Charcot-Marie-Tooth disease (AD, AR)	ND	Childhood to adulthood; Infancy/early childhood	Discussed signs and symptoms of the condition at RoR and will clinically monitor. No family history of symptoms
17	COAGS	18 years	F	DD/NDD, short stature, hirsutism, dsymorphic features	*MITF*	p.Glu425Lys	Susceptibility to cutaneous malignant melanoma (AD)	ND	Adulthood	Discussed surveillance and skin protection at RoR. No family history of melanoma
18	SouthSeq	25 days	F	IUGR, congenital microcephaly, congenital hearing loss, cardiomyopathy, thrombocytopenia, intraabdominal abcess, mid jejunal necrosis/perforation	*PRRT2*	p.Arg217Pfs	Episodic kinesigenic dyskinesia (AD); Benign familial infantile seizures (AD)	ND	Childhood to adolescence	Donor egg; parents did not want findings placed in the EHR but encouraged to share with infant's health care providers; Patient is followed in Neurodevelopmental Clinic and Early Steps
19	SouthSeq	24 days	M	Cardiac and urogenital anomalies	*SCN4A*	p.Ile1455Thr	Paramyotonia congenita (AD)	Pat^a^	Infancy to early childhood	Post RoR, revealed father exhibited symptoms that overlap features reported for *SCN4A-*related condition and was excited to learn of why he has stiffness regularly
20	COAGS	9 months	M	Dysmorphic features, dysphagia, hip dysplasia	*SLC6A5*	p.Cys3Ter	Hyperekplexia (AD, AR)	ND	Newborn to infancy	Family reported no history of symptoms of the condition
21	COAGS	5 years	M	Cutis verticis gyrata, bifid uvula, autism-like behaviors	*SLC7A9*	p.Gly105Arg	Cystinuria (AD, AR)	ND	Childhood to adulthood	Post RoR, reported family history of multiple people with kidney stones. Alerted pediatrician to monitor if symptoms arise

All variants are heterozygous with exception of the missense in ABCD1 (hemizygous)

*M* male, *F* female, *VACTERL* vertebral defects, anal atresia, cardiac defects, tracheoesophageal fistula, renal anomalies, and limb abnormalities, *ID/NDD* intellectual disability, neurodevelopmental delay, *VSD* ventricular septal defect, *FTT* failure to thrive, *AD* autosomal dominant, *AR* autosomal recessive, *XLD* X-linked dominant, *XLR* X-linked recessive, *ND* not determined, *Mat* maternal, *Pat* paternal, *RoR* return of results

^a^Family history of disorder; some not revealed until after RoR

Identified IFs represent a variety of different variant types including missense, nonsense, frameshift, in-frame deletion, splice, and copy number alteration. Most identified IFs are associated with cancer or cardiovascular risk, genitourinary dysfunction, neurological disorders, or muscle tissue disease. Expected symptom onset spans early infancy to adulthood and reported disease penetrance estimates are variable. Five incidental variants were determined to be inherited from a parent with clinical presentation that overlapped features associated with the identified IF (Table 2).

To offer more detailed information pertaining to the types of IFs identified across our four research sites, we below include case summaries that highlight specific incidental findings, discuss their potential for benefit and/or harm, spotlight considerations for return of IFs to pediatric patients (Table 3), underscore associated challenges and opportunities, and demonstrate the potential for IFs to change medical management and improve outcomes.

**Table 3:** Considerations for return of incidental genetic findings to pediatric patients

Indication for testing	Differentiating incidental and primary findings can be difficult in some patients, especially when patients are very young and the age of onset associated with the finding is highly variable. Some identified findings thought to be incidental may represent variation that is associated with the indication for testing and reflects phenotypic expansion or symptom onset that is earlier than previously reported.
Family history	Although the pediatric patient is not exhibiting disease features associated with an incidental finding at time of testing, understanding family history may help determine variant relevance and utility of result return.
Age of onset	Age of disease onset is important to consider in the context of incidental findings. Variants associated with early-onset disease are more likely to alter patient outcome and may be more relevant to younger patients compared to those associated with later-onset disease. Further, as an example, variation associated with disease onset in infancy identified in an adolescent patient may represent variation that is not disease causal or disease with low penetrance.
Penetrance	Return of an incidental finding associated with completely penetrant disease is much more compelling than return of a finding that results in an associated phenotype in only a small proportion of individuals that harbor the genetic finding.
Clinical actionability	Clinical utility and actionability is important to consider in the context of incidental genetic findings. If the finding will alter patient care (e.g., management, treatment) in a way that is beneficial to outcome, then return of the result will be useful to the provider, patient, and family, and is warranted.
Personal utility	It is possible that patients and families receiving incidental genetic findings will find them to be of great personal utility. These types of findings may result in altruistic feelings, enhance self-knowledge and understanding, promote feelings of control, and allow for future planning.
Severity of disease	Incidental findings associated with severe disease phenotypes likely warrant more serious consideration for return. Knowledge about potential onset of severe disease will almost certainly be useful to the provider and patient/family in terms of planning, and may also improve both short and long-term outcomes.

### Patient 1, *ABCD*1

Patient 1 was a male enrolled into SouthSeq at day 14 of life. The patient’s clinical presentation included failure to thrive, hydronephrosis, skin rash, poor feeding, acute kidney injury, developmental dysplasia of the hip, patent foramen ovale, and mild retrognathia.

GS revealed two pathogenic findings in the newborn patient; a pathogenic heterozygous *de novo* variant in *TSC1* (p.Gln413ArgfsTer27; MIM# 605284) that was considered a diagnostic primary finding, and a maternally inherited pathogenic hemizygous variant in *ABCD1* (p.Arg617Cys) that was considered an incidental result at time of GS analysis due to lack of *ABCD1*-associated phenotypes (Table 2). The *ABCD1* variant was discovered using a filtration pipeline aimed at detecting rare and damaging variants (no prioritization based on phenotype). Variation in *ABCD1* is associated with X-linked recessive adrenoleukodystrophy (ALD, MIM# 300100) with onset ranging from childhood to adulthood; childhood onset represents a more severe phenotype. Near complete disease penetrance has been reported in males harboring pathogenic *ABCD1* variation [31]. ABCD1 functions as a transporter molecule and impairment results in the build-up of saturated very long-chain fatty acids (VLCFA) in tissues throughout the body [32]. Clinical diagnosis is typically made through blood testing (VLCFA levels, adrenal insufficiency screening) or MRI. Potential treatments include early-disease-stage bone marrow transplant, steroids, and physical therapy [32].

Based on the likelihood of the newborn developing adrenoleukodystrophy at a later developmental stage, the study team decided to return the result to the patient’s family as an IF, potentially predictive of future symptom onset. At the return of results appointment, the family reported loss of a male family member at a young age that exhibited ALD-like features (nephew of the patient’s maternal great grandmother), potentially suggesting a family history of X-linked ALD. Follow-up testing (post-result return) revealed the infant exhibited increased VLCFA levels suggestive of an adrenoleukodystrophy clinical diagnosis (elevated C26:0 level, 4.13; elevated ratios of C24:0/C22:0 (1.68) and C26:0/C22:0 (0.085); see reference levels described by Rattay and colleagues [33]. Taken together, the *ABCD1* IF was indicative of a potential second underlying genetic condition in the newborn patient and the return of this result empowered the family and clinical team to follow up with additional testing/clinical management (referral to endocrinology and hematology oncology) in an informed manner that may improve both short- and long-term outcomes.

### Patient 18, *PRRT2*

Patient 18 was a female enrolled into SouthSeq at day 25 of life. The patient’s clinical presentation included intrauterine growth restriction (IUGR), small birth length, microcephaly, hearing loss, cardiomyopathy, thrombocytopenia, anemia, meconium ileus, and jejunal necrosis and perforation. The patient was conceived using a donor egg and no information pertaining to the egg donor or her family history was available. GS testing identified no variants that were associated with the indication for testing but did reveal a frameshift variant in *PRRT2* (p.R217Pfs; MIM# 614386). *PRRT2* p.R217Pfs is a well-established pathogenic variant [34, 35] and is the most common variant observed in patients with *PRRT2*-related disorders. This variant was not paternally inherited, although inheritance remains unknown given the biological mother (egg donor) was not available for testing.

Genetic variation in *PRRT2* is associated with benign familial seizures 2 (BFIS2, MIM# 605751) and episodic kinesigenic dyskinesia 1 (EKD1, MIM# 128200). Symptoms of these disorders include seizures and abnormal involuntary movements. Disease onset typically occurs in the first few months of life through adolescence and symptoms remit or become less severe with age [36]. *PRRT2*-related disorders have been associated with incomplete penetrance, result in no lasting neurologic sequelae, and respond favorably to medication [36].

The study decided to return this IF as it seemed medically relevant at the time of testing, and the testing laboratory wanted the provider and family to be aware of this result in case related clinical phenotypes manifest, and since *PRRT2*-related conditions respond well to treatment. While the family was hoping to receive a result describing their infant’s congenital condition, they received a test result suggesting potential future onset of additional symptoms. To further complicate things, the family was counseled that it is possible the variant was inherited from the egg donor.

At the time of the results’ return, the family requested that the *PRRT2* finding not be placed in the electronic health record (EHR), as they were concerned that the result “would make doctors treat the baby differently.” Furthermore, the family was also concerned with incomplete penetrance of the disorder. Without certainty that symptoms would appear, they did not think this information should be included in the medical record. Because of the family’s request, this incidental result was ultimately not added to the infant’s health record, although a hard copy of the IF report was given to the family for future reference.

### Patient 6, *CACNA1A*, *PMP22*

Patient 6 was a male enrolled into SouthSeq at day 19 of life. The patient’s clinical presentation included patent foramen ovale, congenital imperforate anus, and congenital hemivertebrae, suggestive of VACTERL association (vertebral defects, anal atresia, cardiac defects, tracheoesophageal fistula, renal anomalies, and limb abnormalities). GS revealed no genetic variation that would explain the newborn’s congenital issues; however, it did reveal two other variants considered incidental, as they appeared to have no relevance to the newborn’s phenotype at the time of testing. These variants include a pathogenic paternally-inherited heterozygous nonsense variant in *CACNA1A* (p.Arg1545Ter) and a paternally-inherited pathogenic 17p12 deletion (1.33 MB, encompassing *PMP22*).

Genetic variation in *CACNA1A* is associated with autosomal dominant episodic ataxia, type 2 (EA2, MIM# 108500), epileptic encephalopathy (EIEE42, MIM# 617106), migraine (FHM1, MIM# 141500), and spinocerebellar ataxia (SCA6, MIM# 183086), and is defined by incoordination, imbalance, dizziness, migraines, tinnitus, dysarthria, hemiplegia, and muscle cramping. Onset typically occurs in childhood or adolescence and incomplete penetrance has been reported. Symptoms associated with loss-of-function variation may be treated with anti-seizure medication [37] as well as lifestyle and/or dietary changes [38]. Deletion events encompassing *PMP22* are associated with autosomal dominant hereditary neuropathy with liability to pressure palsies (HNPP, MIM# 162500) leading to numbness, tingling and muscle weakness in the limbs. Onset typically spans adolescence to adulthood with near-complete penetrance [39]. Management and/or treatment includes rehabilitation and physical/occupational therapy.

As previously mentioned, both variants were found to be paternally inherited and had implications for not only the newborn but also the father. Because the parent-participants opted into receipt of secondary findings at the time of consent, and because these findings were thought to be of immediate medical relevance, the study team decided to return these two variants to the family as IFs. At results disclosure, the father, who was 20 years old at the time of testing, reported a history of headaches, dizziness, imbalance, abnormal gait, and wrist pain; symptoms that overlap features reported for *CACNA1A*- and *PMP22*-related disorders. While the clinical study team offered the father referral to a neurologist at the return of results, he declined.

### Patient 5, *ATM*

Proband 5 was a previously healthy 4-year-old male diagnosed with standard risk non-WNT/non-SHH medulloblastoma. As part of KidsCanSeq, both ES and focused germline panel testing that included 35 genes associated with childhood cancer predisposition were conducted. Both tests identified a single pathogenic variant in *ATM* (p.D2959Gfs). No additional variants in the *ATM* gene or other variants associated with cancer predisposition were identified by either genetic test. This variant was paternally inherited and the proband’s father was healthy at the time of disclosure. There was no reported paternal family history of cancer (seven healthy aunts, four healthy uncles, both paternal grandparents lived well into their 70s with no cancer).

Per National Comprehensive Cancer Network guidelines (www.nccn.org), variants in *ATM* confer an absolute risk of 15–40% for breast cancer, 5–10% for pancreatic cancer, and <3% for ovarian cancer. Surveillance guidelines are limited to annual mammogram and consideration for breast MRI at 40 years of age. Pancreatic cancer screening is limited to individuals with a family history of pancreatic cancer. There is no clear data on the risk of childhood cancer in individuals carrying a heterozygous *ATM* pathogenic variant and, to date, the *ATM* gene has not been described among the subset of genes associated with germline predisposition to medulloblastoma [40]. Furthermore, biallelic pathogenic variation in *ATM* is associated with ataxia-telangiectasia, which includes progressive ataxia onset between the ages of 1–4 years, oculocutaneous telangiectasias, and increased risk for leukemia and lymphoma [41]. However, there were no clinical concerns for ataxia-telangiectasia in the proband. In this case, the *ATM* finding was related to the indication for testing (broad cancer phenotype) and did not technically meet the definition of an IF set forth in this report, or by the KidsCanSeq study, since it was sought after (cancer panel testing). However, because it was determined to be unlikely that the *ATM* variant explained the proband’s medulloblastoma, and because it demonstrates detection of a genetic finding not associated with the phenotypic indication via panel testing, we believed this case warranted inclusion.

Genetic counseling was provided to the family and no recommendations for additional cancer screening in the proband were initiated. A detailed results letter was shared with the family to assist with communicating the result to other family members for which it would be meaningful. Given the association of *ATM* with adult-onset cancer, genetic testing for the proband’s minor siblings was not recommended at the time of result return; however, the importance of genetic counseling for the siblings later in life was emphasized.

## Discussion

Although the terms sound similar, there are key differences between SFs and IFs. SFs are findings unrelated to the patient’s clinical presentation, but are purposely sought or analyzed as part of genomic testing [1, 3]. While IFs are also unrelated to the indication for testing, they are not actively sought, and in most cases, are unexpected. The ACMG provides guidance on how to approach SFs [3–5]; however, there is no analogous guidance established by US governing bodies for the management of IFs, even though their detection should be anticipated when conducting clinical genomic testing. Laboratories conducting analysis in a phenotype-independent manner are even more likely to detect IFs.

Many IFs share characteristics of genes included on SF gene lists, which can make it hard for laboratories to determine whether findings should be returned. This means individual commercial and research laboratories develop their own policies for IF results, and these policies are often shaped by the emergence of challenging cases. When laboratories adopt different approaches, it adds complexity to pre-test consent and post-test results disclosure, which can become a burden for genetics providers and adversely affect patients.

IFs present a unique challenge for pediatric cases where phenotypic information may be limited based on the patient’s age/developmental stage, leading the laboratory to question if a presumed IF may actually be an as-yet unobserved feature related to the indication for testing. Prior papers addressing the topic of incidental findings in pediatrics have presented both theoretical and experiential arguments [10–12, 42–44]. Here, we add to this knowledge base as we have collected a set of pediatric IFs across several diverse laboratories and clinical sites and describe the utility of these findings (Table 2).

The IFs identified across our studies affect 19 distinct genes and are associated with different conditions exhibiting varying penetrance, severity, and ages of onset. Likely owing at least in part to this variability, IFs were managed across the clinical sites represented in this study in a heterogenous manner. For example, in SouthSeq, patient families were given the option to receive SFs at enrollment, and the study only returned incidentally identified variants if thought be medically actionable. In contrast, COAGS offered patients/families the option to receive a variety of SF/IF categories at time of consent (untreatable childhood disorders, treatable adulthood disorders, untreatable adulthood disorders, carrier of a condition, and pharmacogenomics). As sequencing groups may have different protocols and pipelines that lead to divergence in how IFs are handled, we encourage groups engaged in genomic sequencing — whether research or clinical — to proactively plan for how they intend to characterize what constitutes an IF and the factors relevant to whether to return incidental results to a participant/patient.

The case examples presented in the Results section each reflect different IF challenges in large-scale genomic sequencing of pediatric cohorts. When teams performing GS are aware of these challenges, they can more effectively shape study protocols, test requisition forms, informed consent documents, analytical workflows, and facilitate more effective pre-test counseling. We propose that laboratories carefully consider the items included below in the context of pediatric IFs (Table 3).

### Indication for testing

Differentiating between primary findings and IFs in young patients may be complicated by lack of, or incomplete, phenotypic information at time of testing, making it difficult for laboratories or clinicians to differentiate between primary findings and IFs. Using the example of the *PRRT2* case described above (patient 18, Table 2), at time of enrollment the infant was not reported to exhibit seizures; however, given that the analysis was conducted several months post-enrollment, and given that *PRRT2*-associated phenotypes may manifest within the first few months of life, it is possible that updated phenotype information might result in categorizing this finding as primary instead of incidental.

### Family history

Family history may support relevance and suggest utility of return. While in many cases family history may be limited, may be considered irrelevant to the indication for testing, or unavailable to the testing laboratory, when available it can weigh heavily in the decision pertaining to return. An example we encountered is the case of a neonate harboring paternally inherited IFs affecting *CACNA1A* and *PMP22* (patient 6, Table 2), both associated with neurological phenotypes. Family history information was not available to the testing laboratory at time of analysis, but the neonate’s father was determined to be symptomatic at results disclosure. Having family history information available at time of testing would have made the return of both IFs easier as they provide a potential explanation for the father’s symptoms and highlight the finding’s medical relevance.

### Age of onset

IFs associated with early-onset disease may be more medically relevant for younger pediatric patients. These IFs also present fewer concerns related to the child’s right to make their own decisions about disease-risk awareness than IFs related to adult-onset disease. While we are not suggesting that returning variants associated with later-onset disease is unwarranted or less important, identification/return of variants associated with early childhood and/or adolescent onset disease might improve outcomes and prevent a future diagnostic odyssey or misdiagnosis (see *ABCD1* example in results above).

### Penetrance

Higher disease penetrance may be a more compelling reason to return an IF, but findings associated with conditions with lower penetrance estimates may also warrant return when other factors are considered. Penetrance estimates are difficult to precisely capture, but our knowledge of penetrance for some conditions is expanding with increased comprehensive genomic testing. Across our studies, we encountered IFs in genes associated with variable disease penetrance rates, some exhibiting reported penetrance rates similar to genes included on the ACMG SF genes list. As an example, *CHEK2*-related breast cancer susceptibility is associated with a penetrance rate lower than that reported for *BRCA1/2*, but IFs in *CHEK2* may be just as actionable in terms of cancer screening and surveillance (see NCCN guidelines; www.nccn.org).

### Clinical actionability

Actionability of a specific genomic finding exists on a continuum; it is not binary. Actionability encompasses a spectrum of responses to information that may vary for each individual patient based on their life situation and their access to resources. Actionability for genetic findings are clearest for conditions in which routine screening is possible (even if national guidelines do not exist) and/or medical management is available [45]. Actionability may also encompass overall patient/caregiver awareness for potential risk, which could lead to avoidance of a future diagnostic odyssey (if symptoms arise) or misdiagnosis. While some actionability may include standard-of-care treatments, there is also value to be obtained from findings that could improve proactive screening and surveillance [46]. Moreover, there are some IFs associated with clinical actionability similar to disease genes included on the ACMG SF lists (e.g., *ABCD1*-related adrenoleukodystrophy, patient 1, Table 2). We must also consider actionability as it relates to the potential for future misdiagnosis in patients who become symptomatic. For example, in *HMBS*-related acute intermittent porphyria (patient 13, Table 2), symptoms can evade diagnosis in affected individuals when there are specific preventative measures and acute therapies that can be leveraged. This suggests that the return of this type of result may be warranted despite its low penetrance.

### Personal utility

While it is possible that IFs will result in worry and anxiety (potentially unnecessarily depending on disease severity and penetrance) for patients and their families (see *PRRT2* example in results above), it is also possible that such findings might be of great personal utility. Bunnik and colleagues (2015) propose that genomic information possesses personal utility only if it can be used for decisions, actions, or self-understanding [47]. Returning IFs might result in altruistic feelings, enhance coping, and increase understanding [48]. The knowledge provided with an IF might promote feelings of control and allow patients and families to plan appropriately for the future [47–49]. Moreover, genetics providers anecdotally report that patients and families who receive IFs feel reassured that the study team truly searched for findings related to the indication for testing (primary findings), even if the primary report was negative (novel observation by providers participating in COAGS/SouthSeq). This observation represents an area of psychosocial research that warrants further exploration.

### Severity of disease

Genomic sequencing has the potential to detect IFs associated with diseases that span a wide range of phenotypes with variable severity. IFs associated with severe disease phenotypes potentially resulting in unfavorable outcomes likely warrant more serious consideration for return, compared with findings associated with less severe clinical presentations. Knowledge about potential onset of severe disease will almost certainly be useful to the provider and patient/family in terms of planning, and might also improve both short and long-term outcomes.

## Conclusions

The challenges associated with identification and reporting of IFs is becoming more prominent as patients are sequenced on a larger scale at earlier ages. Given the vast number of potential findings at both the gene and variant level, laboratories may not have the capacity to adjudicate return decisions for each individual finding and may choose to adopt a policy of simply not reporting IFs. However, IFs can greatly impact the lives of patients and their families in considerable ways. In some cases, IFs might lead to unnecessary or needless worry, whereas for other cases they might lead to medical intervention that prevents adverse outcomes. Moreover, IFs will almost certainly burden providers who will need to distinguish the subtle differences between SFs and IFs at time of consent, and who must also manage the downstream clinical activities that result from return. Careful consideration of both benefits and risks must be taken when developing laboratory policies that guide IF detection and return.

The genetics community is quickly expanding knowledge as it relates to phenotypic spectra of disease, clinical variability, estimates of penetrance, new disease genes, and mechanisms of molecular variation – all of which could impact decisions related to IF detection and return. Even among our studies, there were different thresholds for which types of IFs would be returned. Individual laboratories made decisions guided by internal policies and study-specific informed consent, and variants returned by one study with broad return policies may not have been returned by another following more stringent policies.

Furthermore, laboratories must consider how IF-related issues will be managed as technical limitations become less of a barrier, and variant types previously undetected in short-read GS data become visible with newer sequencing technologies that may be employed by testing laboratories in the future (e.g., trinucleotide repeat expansions associated with severe neurological phenotypes detected via long-read sequencing). Clinicians will need to be aware of laboratory policies at the time of test order, as appropriate pre-test consent is crucial. Presumably clinicians are not privy to the behind-the-curtain discussions surrounding IFs that take place on a per case basis, nor are they privy to the considerations that guide decision-making about results return in the laboratory.

Groups may choose to adopt internal policies that govern detection based on the considerations that we highlight above (e.g., actionability, age of onset, and penetrance estimates), but these considerations are subject to interpretation and internal debate could, and likely will, still exist within laboratories. Laboratories are encouraged to adopt a standardized approach to decrease inconsistency when it comes to return of IFs, but we recognize this is almost impossible to articulate in the setting of a constantly growing knowledge base. While the aforementioned cases demonstrate ways in which our laboratories and research studies have managed identified IFs, we recognize other groups may have taken a different approach. This underscores the need and potential benefit for shared guidelines as they relate to identification of IFs in pediatric patients in research and clinical settings. Other groups have also called for standardized approaches and highlighted similar points with reporting incidental findings [50, 51]. Future discussion is required to determine whether cohesive guidelines or policy statements are warranted concerning the detection of IFs both in general and specifically within pediatric patients.

## Supplementary Information

**Additional file 1: Table S1.** Incidental genetic findings identified across four study sites conducting genomic testing for pediatric patients (expanded version of Table 1).

## Data Availability

The genomic data generated for this work are available through dbGaP/AnViL utilizing the study accession numbers: phs002307.v1.p and phs002324.v2.p1. All variants have been submitted to ClinVar (SCV and VCV numbers for all variants are provided in Additional file 1: Table S1).

## Abbreviations

IF: Incidental finding
SF: Secondary finding
ES: Exome sequencing
GS: Genome sequencing
CSER: Clinical Sequencing Evidence-Generating Research Consortium
NHGRI: National Human Genome Research Institute
NCI: National Cancer Institute
NIMHD: National Institute on Minority Health and Health Disparities
SADY: Sequencing Analysis and Diagnostic Yield working group
NICU: neonatal intensive care units
ACMG: American College of Medical Genetics and Genomics
VLCFA: Very long-chain fatty acids
